# Inherent Interfacial Mechanical Gradients in 3D Hydrogels Influence Tumor Cell Behaviors

**DOI:** 10.1371/journal.pone.0035852

**Published:** 2012-04-25

**Authors:** Shreyas S. Rao, Sarah Bentil, Jessica DeJesus, John Larison, Alex Hissong, Rebecca Dupaix, Atom Sarkar, Jessica O. Winter

**Affiliations:** 1 William G. Lowrie Department of Chemical and Biomolecular Engineering, The Ohio State University, Columbus, Ohio, United States of America; 2 Department of Mechanical and Aerospace Engineering, The Ohio State University, Columbus, Ohio, United States of America; 3 Department of Neurological Surgery, The Ohio State University, Columbus, Ohio, United States of America; 4 Department of Biomedical Engineering, The Ohio State University, Columbus, Ohio, United States of America; 5 Department of Neurosurgery and Laboratory for Nanomedicine, Geisinger Health System, Danville, Pennsylvania, United States of America; Consejo Superior de Investigaciones Cientificas, Spain

## Abstract

Cells sense and respond to the rigidity of their microenvironment by altering their morphology and migration behavior. To examine this response, hydrogels with a range of moduli or mechanical gradients have been developed. Here, we show that edge effects inherent in hydrogels supported on rigid substrates also influence cell behavior. A Matrigel hydrogel was supported on a rigid glass substrate, an interface which computational techniques revealed to yield relative stiffening close to the rigid substrate support. To explore the influence of these gradients in 3D, hydrogels of varying Matrigel content were synthesized and the morphology, spreading, actin organization, and migration of glioblastoma multiforme (GBM) tumor cells were examined at the lowest (<50 µm) and highest (>500 µm) gel positions. GBMs adopted bipolar morphologies, displayed actin stress fiber formation, and evidenced fast, mesenchymal migration close to the substrate, whereas away from the interface, they adopted more rounded or ellipsoid morphologies, displayed poor actin architecture, and evidenced slow migration with some amoeboid characteristics. Mechanical gradients produced via edge effects could be observed with other hydrogels and substrates and permit observation of responses to multiple mechanical environments in a single hydrogel. Thus, hydrogel-support edge effects could be used to explore mechanosensitivity in a single 3D hydrogel system and should be considered in 3D hydrogel cell culture systems.

## Introduction

Cell migration is a complex, broad-ranging phenomenon strongly influenced by cues from the external environment such as its chemical nature, topographical architecture, and rigidity [1]. It is now widely appreciated that cells can sense the stiffness of their environment and accordingly alter their response [2]. This was first established in a landmark publication by Pelham and Wang [3], who showed that fibroblasts as well as kidney epithelial cells alter their spreading behavior and motility when plated on substrates with different moduli. Since then, several studies in both two dimensional (2D) and three dimensional (3D) environments have corroborated this finding with other cell types *in vitro* (e.g., neurons [4], endothelial cells [5], myoblasts [6], cancer cells [7]).

Both artificial (e.g., poly(acrylamide) and poly(ethylene glycol)-based systems [8]) and natural (e.g., collagen [9]) polymer hydrogels have been extensively employed to study the effect of cell response to changing substrate rigidity. However, if a single modulus hydrogel is used; several hydrogels are needed to explore effects across a range of mechanical properties. Recently, investigators have begun to incorporate stiffness gradients into hydrogel systems [10]–[18]. These gradients have been shown to better mimic *in vivo* cell response compared to culture in a single modulus mechanical environment [19]. Additionally, they can induce directed cell migration (referred to as “durotaxis” or “mechanotaxis”), in contrast to the random migration that occurs in uniformly rigid microenvironments. However, most of these approaches limit cell culture to 2D, which has been shown to differ from 3D *in vivo* conditions. There are very few studies in 3D exploring the effects of mechanical gradients on cell behaviors [14], [20].

Here, we explore mechanical gradients produced by edge effects at the interface of a rigid support with a soft gel on tumor cell behavior in 3D. Edge effects are a specific engineering phenomenon in which properties of a material alter as a result of interactions with the surrounding medium. This occurs both at the material interface and also in an interfacial boundary region adjacent to the interface. As such, we have examined cell behaviors at a hydrogel-support interface, in a ∼200 µm region surrounding this interface, and as a control, in the bulk of the gel (>500 µm from the rigid support). We speculated the existence of these gradients in hydrogels, as softer gels (e.g., Matrigel, modulus ∼450 Pa [21]) are usually supported on rigid plastic/glass tissue culture plates (modulus of glass, plastic >100,000 Pa [22]), providing a sharp interface between mechanical moduli. We performed finite-element analyses to support our expectation of the presence of these gradients. We then explored the response of glioblastoma multiforme (GBM) cells, a type of brain cancer previously shown to be sensitive to stiffness in 2D [23] and 3D [24], in this system, characterizing cell spreading and morphology, intracellular actin organization, and migration capacity. As a model, we used Matrigel hydrogels, which have previously been employed as a substrate to study GBMs in the traditional transwell insert assay and as individual gels [25], [26], supported on glass. These inherent stiffness gradients do not require external devices or alteration of ligand density or chemical composition and could easily be expanded to other hydrogels and substrates. Further, to our knowledge, this is one of the first studies to show that inherent interfacial mechanical environments in a single, 3D hydrogel influence tumor cell response.

## Materials and Methods

### Ethics Statement

The Ohio State University's Institutional Review Board approved this study under IRB protocol 2005C0075 (dated November 7, 2008). Written consent from all participants in the study was obtained in accordance with the protocol.

### Modeling

A Finite Element Model (FEM) was created using ABAQUS CAE 6.8-1 software (Dassault Systèmes Simulia Corporation, Providence, RI, 2008). This model focused on the mechanical environment exhibited by 100% v/v Matrigel in the vicinity of the bottom of a cell culture well plate having a diameter of ∼7 mm. The sides of the gel were free and without curvature at the bottom thus forming a cylinder. The model simulated an indentation test where a steel indenter tool was used to compress the top of Matrigel with heights of 12.5 µm, 25 µm, 50 µm, 100 µm, and 200 µm. The tip of the indentation tool was spherical and had a diameter of 10 µm, which is approximately the same diameter as the cells being studied. Following the Matrigel indentation, the mechanical properties were obtained. The effective stiffness was determined by fitting a straight line to the force-deflection curve. An axisymmetric model captured the cylindrical geometry of the Matrigel and spherical indenter. The bottom of the Matrigel was fixed mechanically to simulate the hard substrate at the well bottom, by preventing the nodes of the elements from horizontal and vertical translation. Finally, modeling results for heights >200 µm are not included, as additional changes in height did not influence results.

The following assumptions were made for the model:

- The Matrigel was assumed to be elastic and isotropic with an elastic modulus of 450 Pa [21]. A linear elastic model was chosen for simplicity. It is recognized that Matrigel may in fact exhibit viscoelastic properties; however, because the model examines effective stiffness (i.e., instantaneous elastic response), the material will appear stiffer near the rigid support regardless of the model chosen.
- The contact surface between the indenter and Matrigel was assumed to be frictionless. Cells interact with gels via both compression and tension forces. Compression was chosen for this model as this is the least sensitive to the adhesion between the Matrigel and glass. However, results using a tension or shear model would be qualitatively similar.

### OSU-2 Cell Isolation and *In Vitro* Cell Culture

**Figure 1:**
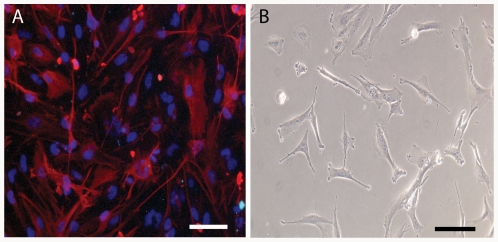
OSU-2 cells in culture. (A) Hoechst stain labels the nucleus blue; rhodamine-GFAP (e.g., glial fibrillary acidic protein) labels the cytoskeleton. GFAP is an intermediate protein expressed by astrocytes. GFAP staining was performed to confirm astrocytic lineage. (B) Phase contrast image of OSU-2 cells in culture. Scale bar indicates 100 µm.

OSU-2 cells were isolated from a GBM patient at the Ohio State University under human IRB protocol 2005C0075. Briefly, tumors were washed with media containing 200 units penicillin, 200 µg streptomycin, and 0.5 µg/ml amphotericin B (all from Invitrogen). Tumor samples were then subjected to 200 units/ml type 1A collagenase (Sigma) for ∼4 hours, triturated, centrifuged at 250 g (∼5 min), and resuspended in cell culture media (DMEM/F12, Invitrogen) containing 10% fetal bovine serum (Invitrogen), 100 units penicillin, 100 µg streptomycin, 0.25 µg/ml amphotericin B. Cells were cultured in a 37°C, 5% CO_2_ environment, fed 2–3 times weekly, and passaged on reaching confluency. Histopathology at the time of operation confirmed tumor type and grade (data not shown) and to further confirm their astrocyte lineage, OSU-2 cells were stained with the glial fibrillary acidic protein (GFAP) marker (Figure 1).

### OSU-2 Cell Seeding in BD Matrigel

To encapsulate cells in BD Matrigel (BD Biosciences), OSU-2 cells were pre-labeled with Cell Tracker Green CMFDA (Invitrogen), suspended in cell culture medium, and mixed at ∼3000 cells/80 µL hydrogel with ice-cold Matrigel at varying concentrations (40, 55, 70, 85 v/v %) in an ice bath. Constructs were incubated at 37°C, 5% CO_2_ for ∼0.5 hours prior to addition of additional OSU-2 cell culture media to encapsulate the cells. Cells were also seeded on BD Matrigel in 2D after the initial gelation of Matrigel constructs at similar concentrations. All Matrigel constructs were prepared in 16-well Lab-Tek chamber slides (Thermo scientific).

### OSU-2 Morphology and Cell Spreading Characterization in 3D Matrigel

OSU-2 laden Matrigel constructs were prepared as described above. After ∼16 hours, still images were captured from each gel at different gel heights using an inverted microscope (Olympus IX71) (N = 3 hydrogels for every formulation) equipped with a spinning disk confocal attachment and a Photometrics Evolve EMCCD camera. Data were subjected to image analysis using NIH ImageJ image analysis software. Discrete cells that were in focus in each image were analyzed to obtain cell area and aspect ratio at different gel heights. Cell areas and aspect ratios versus height are reported as average ± S.D. for total cells found at a particular gel height. Cell areas at the lowest and highest gel positions (images obtained using a confocal microscope (LSM 510; Zeiss, Minneapolis, MN)) were compared and analyzed for statistical differences. Because of variations in surface roughness, cell height was measured from the first plane of observed cells. Thus, the zero point of each chart is equivalent to the lowest plane in which cells were observed and not necessarily the bottom of the substrate. To further quantify cell position, confocal Z-stacks were collected and prepared using the ImageJ Volume Viewer Plugin (metadata available on request).

### Immunostaining for Actin in 3D Matrigel

OSU-2 cells were seeded in Matrigel constructs as described above. After ∼16 hours, cell-gel constructs were fixed in 4 wt/v% paraformaldehyde (Sigma) for 20 min, washed with phosphate buffer saline (PBS), extracted with Triton X-100 (Sigma) solution for 15 min, and blocked with bovine serum albumin (BSA) (Jackson ImmunoResearch) solution overnight at 4°C. Constructs were then incubated with Alexa Fluor® 633 phalloidin (Invitrogen) overnight at 4°C and imaged using fluorescence microscopy to observe actin distribution in 3D Matrigel constructs.

### Real time Cell Tracking in 3D Matrigel

OSU-2 cells (∼5000 cells/well) pre-labeled with Cell Tracker Green CMFDA (Invitrogen) were encapsulated in varying Matrigel concentrations as described above. OSU-2 cell migration experiments were performed using a confocal microscope (LSM 510; Zeiss, Minneapolis, MN) equipped with a weather station to maintain a 37°C, 5% CO_2_ environment. After ∼16 hours, a series of still images of cells in the lowest and highest planes (difference in lowest and highest plane heights ≥∼900 µm) inside the gels were captured every 20 minutes for 12 hours. These images were then concatenated and converted to movies using NIH Image J and were subsequently tracked using the M-Track J plug-in. At least 15 individual cells were tracked at the lowest and highest gel positions (N = 3 hydrogels per condition). In most cases, considerable gel movement (i.e., swelling) was observed as the experiment progressed. This was corrected using the StackReg plugin (available at http://bigwww.epfl.ch/thevenaz/stackreg/) that permitted stack alignment at different time points. Migration speeds were then computed for individual cells by dividing the total length of movement by the observation time and are reported as average ± SD for the lowest and highest gel planes examined per condition.

### Statistical Analysis

Statistical analysis was performed using JMP software (Version 9). All measurements at the highest and lowest gel positions were compared using ANOVA and the Student's t- test.

## Results

### Modeling the substrate/gel interface

Recognizing that there are often edge effects at the interface of two materials with dramatically different moduli, we hypothesized that hydrogels supported by rigid substrates (i.e., glass or tissue culture plates) would display edge effects in their mechanical moduli near the substrate interface. We further hypothesized that these inherent mechanical gradients might influence cell behavior in a single, 3D hydrogel construct. To determine the potential extent of edge effects, we modeled the substrate-hydrogel interface for Matrigel, a common hydrogel biomaterial, on glass using a finite element model. Because it would be difficult to measure forces directly within 3D gels, FEM was employed on gels of different heights to imitate different z positions within a thick 3D gel. However, the cell-laden gels used for experimentation were of the same height (∼2 mm) with cells occupying different positions within the gel.

**Figure 2:**
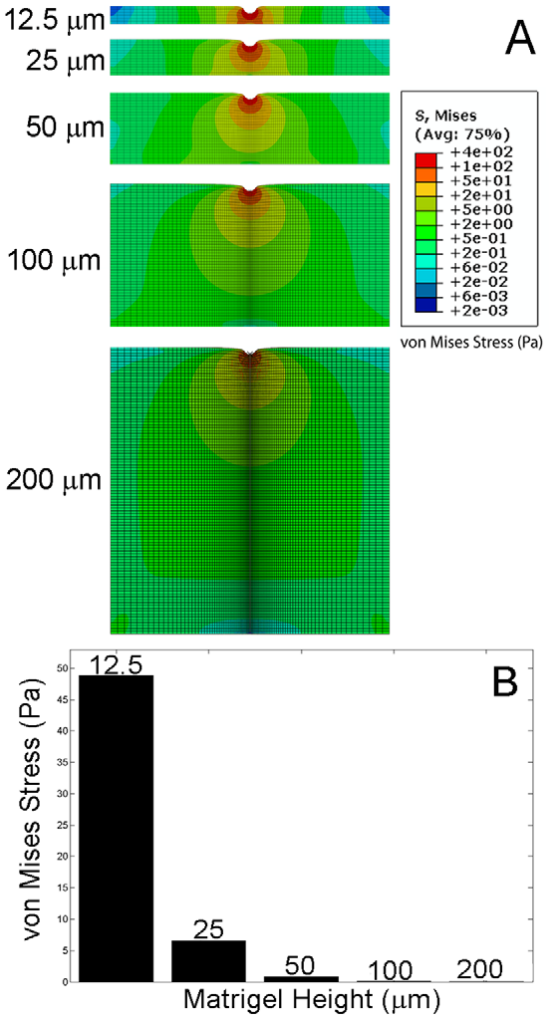
Mechanics of the gel-glass interface modeled using FEM. (A) Stress contour plots of Matrigel with varying height. Axisymmetric elements used. Von Mises stress is an equivalent stress that includes both normal stress (tension/compression) and shear stress contributions. It is calculated from the stress components acting at each location and gives a convenient way of comparing the overall magnitude of stress in different regions. (B) Stress felt at the Matrigel-glass interface as a function of gel height.

In each simulation, the indenter was displaced by 5 µm and the resulting force and stress contour were obtained (Figure 2). As Matrigel thickness approached the size of the indenter tip, which represents the size of the cell, the maximum stress in the gel increased. For Matrigel samples with heights greater than 50 µm, the stress field around the indenter did not interact with the rigid well bottom, whereas for heights less than 50 µm the stress field did interact with the well bottom. Consequently, decreasing the gel thickness led to a stiffer response, since the indenter (i.e., cell) started to feel the effects of the rigid substrate beneath the Matrigel.

**Figure 3:**
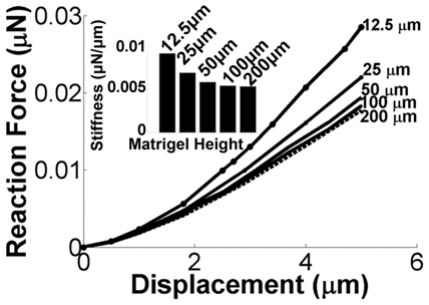
Reaction force vs. 5 µm displacement of the indenter. Insert illustrates a decrease in stiffness with increasing Matrigel height due to a 5 µm indenter displacement. The stiffness insert is the slope extracted from the displacement vs. reaction force plot using the reaction force experienced by Matrigel when the indenter reaches a displacement of 5 µm.

The influence of gel height on stiffness was also examined using the slope generated by plotting the reaction force experienced by the Matrigel due to the indenter displacement (Figure 3). The stiffness of the Matrigel is its resistance to deformation due to an applied force, represented by the slope of the curves in Figure 3. The stiffness of the Matrigel decreased as the thickness of the Matrigel increased (Figure 3), as evidenced by the decrease in slope of the reaction force versus displacement curve. Changes in stiffness were more dramatic for smaller thicknesses of Matrigel (i.e., <50 µm), whereas at heights >50 µm changes in stiffness with increasing gel height were negligible.

### OSU-2 Cell Spreading in 3D Matrigel

To examine the influence of inherent interfacial mechanical gradients on cell behavior, OSU-2 cells encapsulated in Matrigel were analyzed for cell spreading area and aspect ratio. Because of variations in surface roughness, zero height was normalized to the first plane in which cells were observed. To further verify cell position, confocal imaging was performed. Z-stack 3D views of cells in interfacial regions (e.g., ∼0–50 µm and 0–100 µm) are shown in Figures S1 and S2 taken from Stacks S1 and S2, respectively. These stacks and images indicate that cells at positions lower than ∼15 µm most likely make some contact with the rigid support, whereas cells at positions above ∼30 µm are most probably fully embedded in hydrogel. However, cells at the lowest observation plane (i.e., position 0) demonstrated a distinct morphology from those cultured on 2D rigid supports. These cells displayed mostly spindle-shaped morphologies with large processes versus those on bare 2D glass surfaces, which evidenced mostly fan- or tear drop-shaped morphologies (Video S1 vs. Video S9). Also, calculation of individual cell aspect ratios (ratio of major to minor axis of a single cell by fitting an ellipse) showed that cells at the lowest position had statistically higher aspect ratios compared to those plated on glass (Figure S3, 40% (v/v) Matrigel, data for 55%, 70% and 85% (v/v) compositions are also significantly higher compared to glass, not shown).

**Figure 4:**
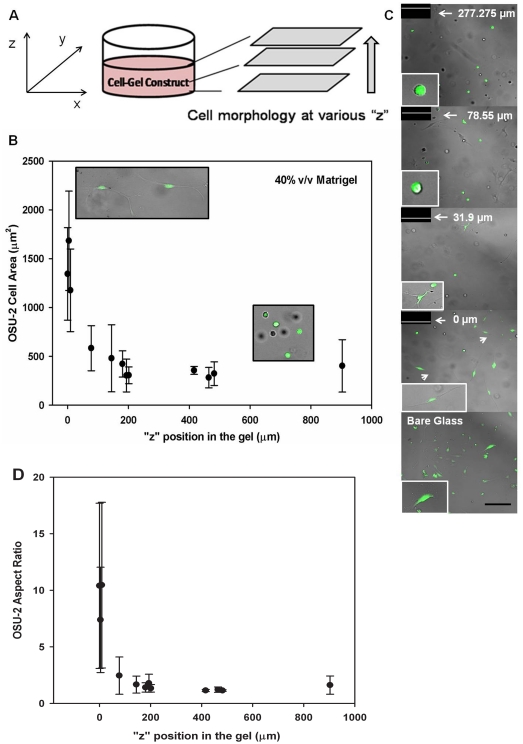
OSU-2 cell behaviors as a function of observation plane in 40% (v/v) Matrigel. (A) Schematic of cell-hydrogel constructs showing morphology observation at different “z” planes. (B) OSU-2 cell area. Representative cell morphologies are shown in the insets. As a result of surface roughness, zero height was set to the first plane of observed cells, which may not necessarily correspond to the substrate surface. (C) OSU-2 morphology at different heights. Representative heights are shown in the chart. Scale bar = 200 µm. (D) OSU-2 aspect ratio.

Morphology in 3D gels varied with changing gel height. For example, cells near the lowest observation plane (i.e., <∼50 µm) showed more elongated, highly bipolar morphologies, whereas cells at higher observation planes (i.e., >∼500 µm) showed rounded morphologies with short processes in some cases (Figure 4). [Still images, with their observations planes, from a typical experiment of cells encapsulated in 40% v/v Matrigel are shown in Figure 4C]. This behavior was quantified as a function of observation plane (Figure 4, S4, S5, and S6) with OSU-2 cells displaying drastically reduced cell area as well as aspect ratio as distance from the lowest observation plane increased for all gel formulations investigated. For instance, OSU-2 cells encapsulated in 40% v/v Matrigel at the lowest observation plane displayed an average cell area of ∼1340±470 µm^2^ (aspect ratio ∼10.4±7.3) versus cells at the highest position investigated displaying an average area of ∼400±270 µm^2^ (aspect ratio ∼1.6±0.8). In comparison, the area of cells cultured on bare glass was 2309±1232 µm^2^ (aspect ratio ∼2.2±1.5), distinct from observations in both higher and lower positions. Also, deviations in cell area measurements reduced with increasing distance from the lowest observation plane. The large deviations observed at low observation planes are a result of the presence of two populations of cells (Figure 4): cells displaying spindle-shaped spread morphology and rounded cells, whereas at more distant observation planes, cells were primarily rounded. Statistical analysis (Student's t-test as well as non-parametric data comparison using Wilcoxon method) confirmed that cell areas for highest and lowest observation planes were statistically significant for all formulations (p = 0.0003 for 40% v/v and 55% v/v, p = 0.0005 for 70% v/v, p = 0.0001 for 85% v/v reported for the Wilcoxon method). For comparison, we also examined cells plated on 2D Matrigel (i.e., cultured on top of Matrigel surfaces), which behaved similar to those at the highest 3D gel positions for all gel formulations (Figure S7).

### OSU-2 Intracellular Morphology in 3D Matrigel (Actin Organization)

**Figure 5:**
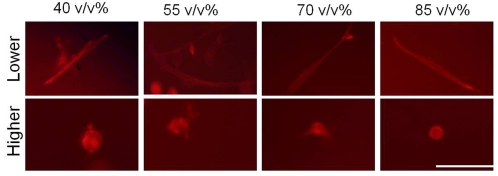
OSU-2 actin organization in 3D hydrogels at a higher observation (>∼500 µm) and lower observation (<∼50 µm) plane. Scale bar = 100 µm.

To further evaluate the influence of inherent mechanical gradients on cell behavior, we also examined actin organization in cells as a function of gel position. Consistent with OSU-2 cell morphology observations, actin filaments in OSU-2 cells at the lowest observation plane were highly organized and resulted in the formation of mature stress fibers. In contrast, cells at higher observation planes, displayed actin fibers that were not organized, poorly developed and did not display stress fiber formation (Figure 5). This behavior was also maintained for cells plated on 2D Matrigel (data not shown).

### OSU-2 Cell Migration in 3D Matrigel

**Figure 6:**
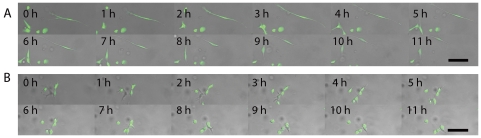
OSU-2 cell migration in a representative 40% v/v Matrigel at (A) lower (<∼50 µm) and (B) higher (>∼500 µm) observation planes shown as stills from time lapse microscopy. Time stamp is reported in hours (h). Scale bar = 100 µm.

**Figure 7:**
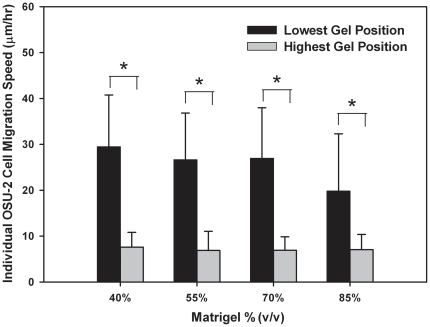
Quantification of migration speeds (average) of OSU-2 cells at the lowest (<∼50 µm) and highest observation planes (>∼500 µm) investigated. * indicates statistical significance.

OSU-2 cells encapsulated in Matrigel were tracked in real time to gain insight into their migration patterns as well as to quantify migration speeds. There were striking differences in migration speeds and patterns of OSU-2 cells at the lowest observation plane, where edge effects were expected to dominate, compared to those near the gel surface. For example, in the case of 40% v/v Matrigel, cells at the lowest observation plane migrated at ∼29.5±11.3 µm/hr, ∼4× faster than cells near the gel surface that migrated at ∼7.6±3.1 µm/hr. This trend was maintained at all concentrations of Matrigel investigated. OSU-2 cells at lower observation planes migrated by displaying highly bipolar cell bodies and long processes (see videos S1, S3, S5 and S7), whereas cells at higher gel positions migrated by displaying short processes and mostly rounded or ellipsoid cell bodies (see videos S2, S4, S6, S8). [Migration stills from a typical time lapse experiment for cells at lower and higher observation planes in the 40% v/v gel are shown in Figure 6.] Migration speeds for each gel formulation were computed for the lowest and highest observation planes investigated and were compared with cells plated on the bottom of glass surface controls using ANOVA and the Student's t-test. In all gel formulations, statistically significant differences (p<0.001 in all cases) were detected for cell migration speeds at lower vs. higher observation planes (Figure 7). Migration speeds of cells at lower observation planes were not statistically significant from those of cells on glass substrates (video S9) (OSU-2 cell speed = 28.2±9.7 µm/hr). It is likely that cells at the “zero” observation plane are in contact with the glass surface; however, sequential data collected from cells throughout the 0–200 µm interfacial region (e.g., Figure 4B, S1, S2, Stack S1, Stack S2) clearly demonstrates that cell area decreases with gel height, supporting the hypothesis that interfacial gradients are influencing cell behavior.

## Discussion

Mechanical gradients that exist *in vivo* (e.g., in the brain) have been shown to modulate cell migration, differentiation, proliferation, and cytoskeletal organization [19]. We hypothesized the existence of mechanical gradients at the interface of soft hydrogel materials and rigid substrate supports that could influence cell behavior. To support our expectation of the presence of these gradients, FEM was used to simulate an indentation test on 100% v/v Matrigel supported on glass. Other computational models that examine similar interfaces focus on the cell and gel simultaneously [27], [28]. Despite the fact that our simplified FEM does not consider either a 2D [28] or 3D matrix [27] in contact with cells, our results corroborate those of more complex models. FEM shows that the stiffness exhibited by Matrigel changes as the Matrigel depth approaches the size of the cells. Specifically, the model shows an increase in stiffness for Matrigel heights ≤50 µm from the hard substrate. This increased stiffness yields an increase in Matrigel stresses near the well bottom. In addition to FEM, active microrheology could be further employed to experimentally support simulation observations. In particular, this could be achieved by embedding micro beads in 3D hydrogels and tracking their response to external fields (e.g., using magnetic fields, or optical tweezers) at different gel heights. This could then be translated to appropriate stress-strain relationships and mechanical properties.

Experimentally, GBM tumor cells responded to this increased stiffness by exhibiting a spread or bipolar morphology. Morphological changes exhibited an exponential response (Figure 4), decaying with increasing distance from the rigid support. Cells also displayed an increased migration capacity, in stark contrast to cells distant from the interface. This outcome is in agreement with recent studies, which show that the stiffness of the gel and substrate are crucial factors affecting cell morphology during migration [7], [10], [29]. This was also evident from individual cell area and aspect ratio analyses in which cells at lower observation planes exhibited higher and statistically significant cell areas and aspect ratios compared to cells more distant from the interface. Thus, these data show that inherent gradients in 3D culture systems can dramatically influence the ability of cells to attach and spread, and demonstrate that tumor cells encapsulated in 3D hydrogels can “sense” the stiffness of an underlying rigid support (in this case, glass).

This phenomenon has been previously observed by Discher and colleagues in 2D gels for mesenchymal stem cells [22], [30] and others [31]–[33]. A similar result has also been obtained computationally by van Dommelen et al. [34], who showed that glass plates used to support brain tissue samples played a significant role on the force level in indentation. Our simulations and experimental findings complement these studies by examining tumor cell behaviors in a 3D setting. Our findings also suggest caution in interpretation of cellular outcomes in 3D hydrogels depending upon the location of these cells in hydrogels, as edge effects can significantly alter findings. Further, individual cells can influence the behavior of neighboring cells several microns distant [29], and hence the influence of edge effects could extend beyond the interfacial region. Our findings are consistent with a recent study that demonstrated that an underlying rigid support dramatically influences human mesenchymal stem fate when cultured on 2D collagen gels of different heights (i.e., 130 µm versus 1440 µm) [35]. Several experimental and computational studies have attempted to identify a “critical” height that defines how far cells can sense microenvironments in 2D [22], [28], [31]–[33]. Our computational findings suggest a possible threshold of ∼50 µm for a 3D Matrigel model. This is comparable to values obtained in previous studies, which yield “critical” height values that range from on the order of focal adhesions (i.e., a few microns) [22], [28], [32] to ∼2–3 times the cell length (e.g., ∼60 microns) [31], [33].

In addition to changes in cell spreading and morphology, we also observed differences in cell migration at the lowest and the highest observation planes. Individual cancer cells can migrate in a mesenchymal or amoeboid fashion in 3D matrices [36]. In mesenchymal migration, cells attach to the matrix via formation of focal contacts that are dissolved during migration [36]. Cells migrating in amoeboid mode squeeze their cell body through matrix pores with minimal matrix contact or no attachment [36]. OSU-2 cells at lower observation planes migrated faster (∼4×) and in a mesenchymal fashion with filopodia (finger like protrusions) at the leading cell edges (e.g., Video S1). In contrast, cells more distant from the rigid support showed continuous short process extension and retraction (e.g., Video S2). Some cells seem to migrate in an amoeboid fashion (e.g., Video S6); however, this was not consistently maintained for all cells. Differences in migration modes were further supported by data on actin organization. Cells at lower observation planes displayed highly organized stress fiber formation, which enables cells to generate traction forces for migration, in contrast to cells more distant from this interface. These results demonstrate that tumor cells may adopt different migration mechanisms in a single 3D hydrogel in response to these inherent interfacial mechanical gradients.

Interestingly, no specific trends in cell behaviors (i.e., cell spreading, migration, actin organization) were observed with increasing Matrigel concentration (e.g., Figure 7). Thus, the influence of edge effects observed here was more significant than that of the increased modulus generated by increasing Matrigel concentration. Across the concentration ranges investigated, Matrigel demonstrates an ∼5 fold difference in stiffness [7], whereas interfacial gradients may access as much as a 10 fold increase in stiffness (e.g., Figure 2). Furthermore, cells cultured in single gels far from the substrate will experience a uniform mechanical environment, as opposed to gradients, such as those found near the interface with a support. Gradients can produce different cell responses than uniform mechanical environments [10]–[20]. Also, it should be noted that additional variables, such as matrix porosity, which would differ in gradient and single gel systems, may have influenced results. Thus, it is not surprising that the responses seen with increasing gel concentration differ from those produced by interfacial gradients.

There are several factors that may influence these results, which could be minimized by experimental modifications. The addition of fluorescent beads as markers of gel position or the use of computational algorithms to reduce gel movement would potentially permit tracking throughout the gel over longer time frames. Additionally, dynamic investigations of cell behaviors immediately after cell encapsulation should provide insight into the metabolic rates of tumor cells that further relate to differential cell spreading and migration behaviors. It should also be noted that a possible limitation of this approach is that different conditions in the bulk vs. at the gel-support interface (e.g., nutrient supply, ligand or crosslinker density) could lead to differences in properties that influence cell behaviors. The correlation between interfacial stiffness and pore size at different gel heights should be experimentally explored in detail. This will enable examination of complex relationships of matrix parameters (i.e., pore size) on tumor cell migration in 3D microenvironments.

Here, we demonstrate that inherent interfacial mechanical gradients produced by edge effects between soft hydrogels and rigid substrate supports can modulate cell behavior. To our knowledge, this study is the first to examine the role of inherent interfacial mechanical gradients on the behavior of tumor cells in a single 3D hydrogel. These findings are broadly applicable to virtually any hydrogel and adhesive rigid support combination, and could have import for hydrogel-based, 3D cell culture. Thus, inherent mechanical gradients can influence cell behavior in single, 3D hydrogels.

## Supporting Information

Figure S1 Still images taken from a Z-stack of fluorescently-labeled cells in a 40% v/v Matrigel (0–50 µm, step size = 5 µm). (A) Brightfield/fluorescence Z-stack shown as a montage. (B) Rotated views of the Z-stack shown in A. White arrow indicates the same cell, at position 30 µm, which is clearly embedded within the hydrogel.
(TIF)

Figure S2 Images from a brightfield/fluorescence Z-stack of fluorescently-labeled cells in a 40% v/v Matrigel (0–100 µm, step size = 5 µm). White arrow indicates a cell, at position 15 µm, whose edge is in contact with the rigid glass support while the cell body is embedded in the hydrogel. The asterisk indicates a cell, at position 90 µm, fully embedded in the hydrogel.
(TIF)

Figure S3 Box plot of individual cell aspect ratios comparing cells in the lowest observation plane (<∼50 µm) in 40% (v/v) Matrigel versus Bare Glass. * indicates statistical significance (p<0.0001), n = 206 cells for glass, n = 20 for lowest observation plane in 40% (v/v) Matrigel.
(TIF)

Figure S4 OSU-2 cell morphology quantification. (A) OSU-2 cell area and (B) aspect ratio as a function of observation plane in 55% (v/v) Matrigel.
(TIF)

Figure S5 OSU-2 cell morphology quantification. (A) OSU-2 cell area and (B) aspect ratio as a function of observation plane in 70% (v/v) Matrigel.
(TIF)

Figure S6 OSU-2 cell morphology quantification. (A) OSU-2 cell area and (B) aspect ratio as a function of observation plane in 85% (v/v) Matrigel.
(TIF)

Figure S7 OSU-2 cell morphology in 2D Matrigel for all formulations. Scale bar = 200 µm.
(TIF)

Stack S1 Brightfield/fluorescence Z-stack of fluorescently-labeled cells in a 40% v/v Matrigel (0–50 µm, step size = 5 µm).
(AVI)

Stack S2 Brightfield/fluorescence Z-stack of fluorescently-labeled cells in a 40% v/v Matrigel (0–100 µm, step size = 5 µm).
(AVI)

Video S1 OSU-2 cell migration at a lower observation plane (<∼50 µm) in 40% v/v Matrigel.
(AVI)

Video S2 OSU-2 cell migration at a higher observation plane (>∼500 µm) in 40% v/v Matrigel.
(AVI)

Video S3 OSU-2 cell migration at a lower observation plane (<∼50 µm) in 55% v/v Matrigel.
(AVI)

Video S4 OSU-2 cell migration at a higher observation plane (>∼500 µm) in 55% v/v Matrigel.
(AVI)

Video S5 OSU-2 cell migration at a lower observation plane (<∼50 µm) in 70% v/v Matrigel.
(AVI)

Video S6 OSU-2 cell migration at a higher observation plane (>∼500 µm) in 70% v/v Matrigel.
(AVI)

Video S7 OSU-2 cell migration at a lower observation plane in (<∼50 µm) 85% v/v Matrigel.
(AVI)

Video S8 OSU-2 cell migration at a higher observation plane (>∼500 µm) in 85% v/v Matrigel.
(AVI)

Video S9 OSU-2 cell migration on a glass substrate. Note the fan-like morphologies exhibited in traditional 2D cultures.
(AVI)
