# Supplementary figures and images for: Inherent Interfacial Mechanical Gradients in 3D Hydrogels Influence Tumor Cell Behaviors
Source: PLoS One. 2012 Apr 25;7(4):e35852. doi: 10.1371/journal.pone.0035852 (PMC3338483; doi:10.1371/journal.pone.0035852)

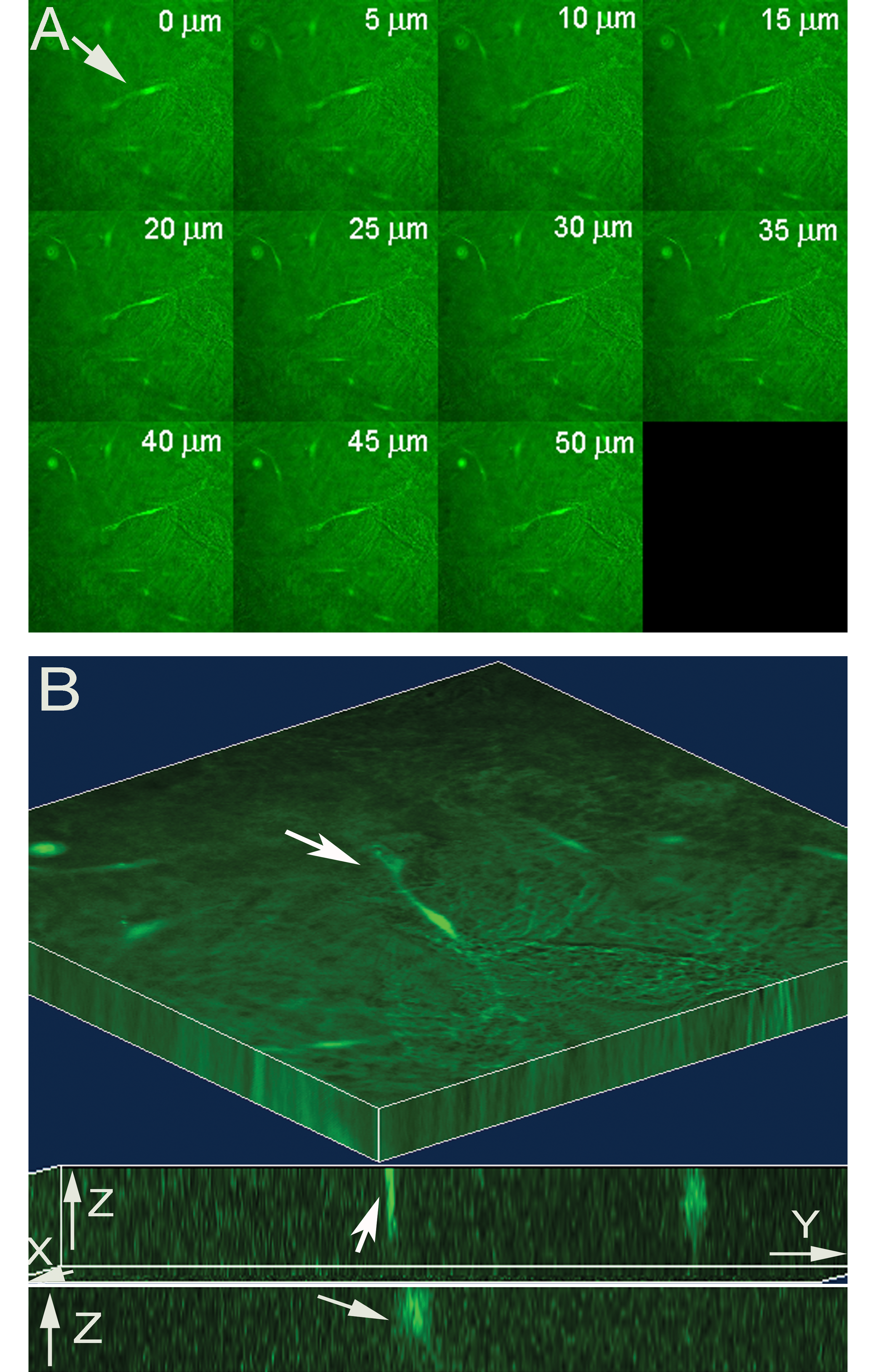

Supplement: Figure S1 — Still images taken from a Z-stack of fluorescently-labeled cells in a 40% v/v Matrigel (0–50 µm, step size = 5 µm). (A) Brightfield/fluorescence Z-stack shown as a montage. (B) Rotated views of the Z-stack shown in A. White arrow indicates the same cell, at position 30 µm, which is clearly embedded within the hydrogel. (TIF) [file pone.0035852.s001.tif]

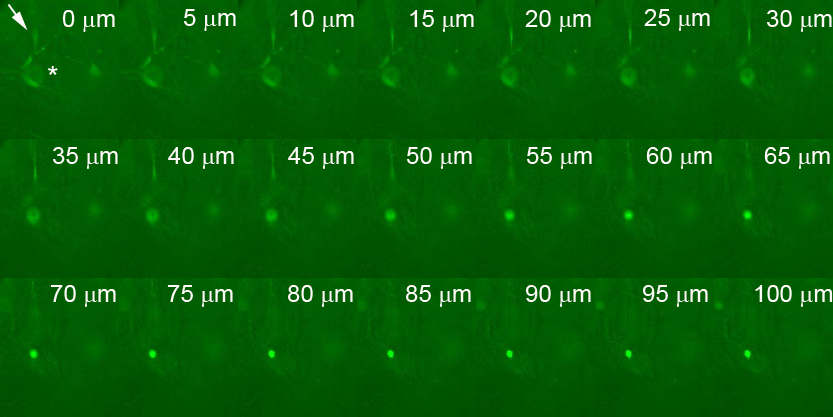

Supplement: Figure S2 — Images from a brightfield/fluorescence Z-stack of fluorescently-labeled cells in a 40% v/v Matrigel (0–100 µm, step size = 5 µm). White arrow indicates a cell, at position 15 µm, whose edge is in contact with the rigid glass support while the cell body is embedded in the hydrogel. The asterisk indicates a cell, at position 90 µm, fully embedded in the hydrogel. (TIF) [file pone.0035852.s002.tif]

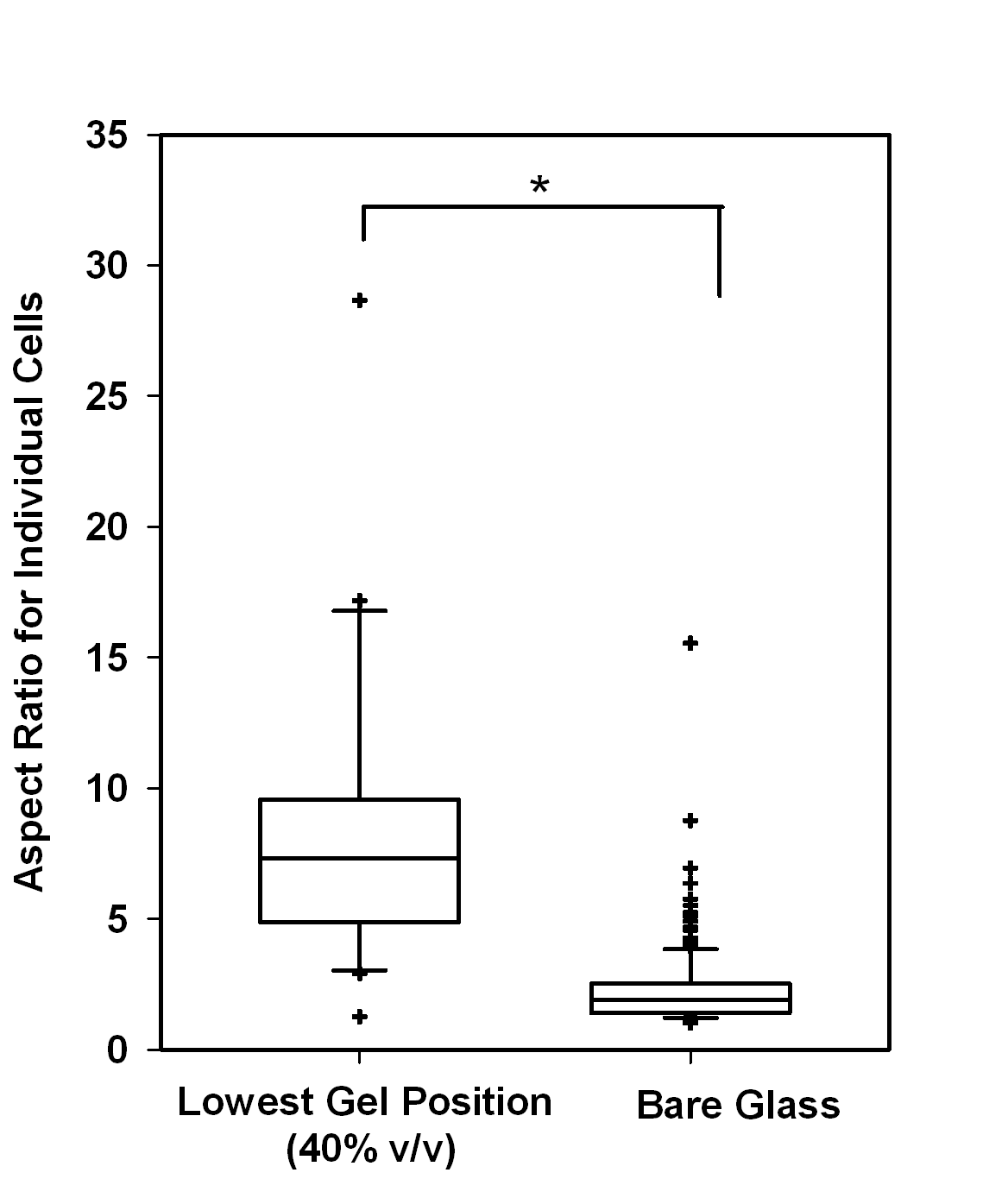

Supplement: Figure S3 — Box plot of individual cell aspect ratios comparing cells in the lowest observation plane (<∼50 µm) in 40% (v/v) Matrigel versus Bare Glass. * indicates statistical significance (p<0.0001), n = 206 cells for glass, n = 20 for lowest observation plane in 40% (v/v) Matrigel. (TIF) [file pone.0035852.s003.tif]

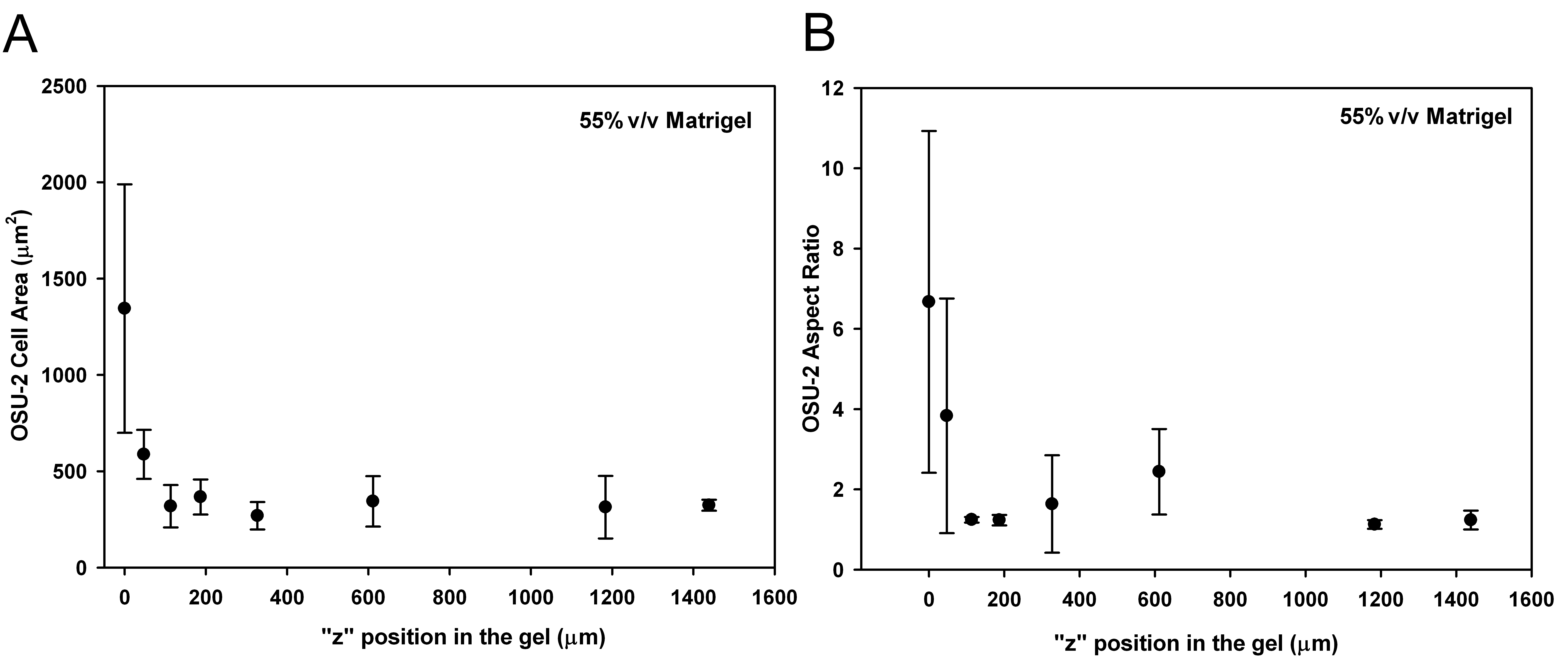

Supplement: Figure S4 — OSU-2 cell morphology quantification. (A) OSU-2 cell area and (B) aspect ratio as a function of observation plane in 55% (v/v) Matrigel. (TIF) [file pone.0035852.s004.tif]

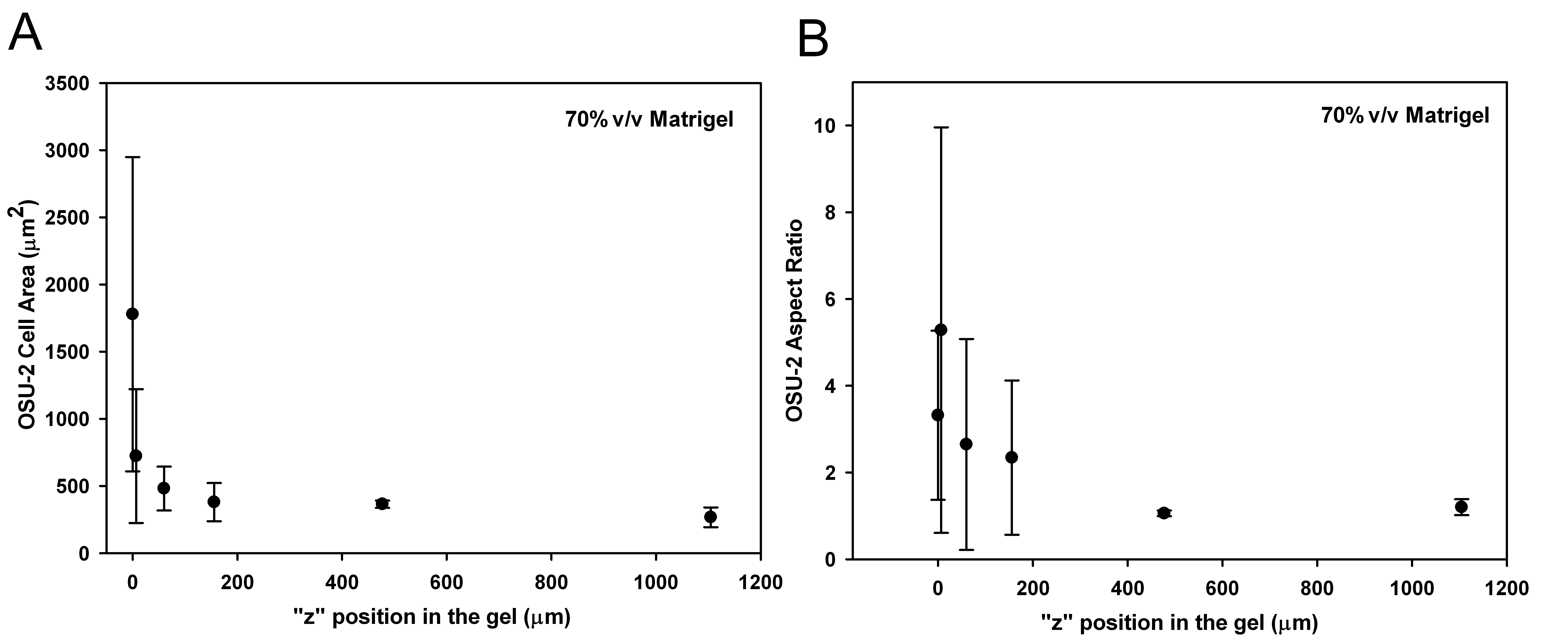

Supplement: Figure S5 — OSU-2 cell morphology quantification. (A) OSU-2 cell area and (B) aspect ratio as a function of observation plane in 70% (v/v) Matrigel. (TIF) [file pone.0035852.s005.tif]

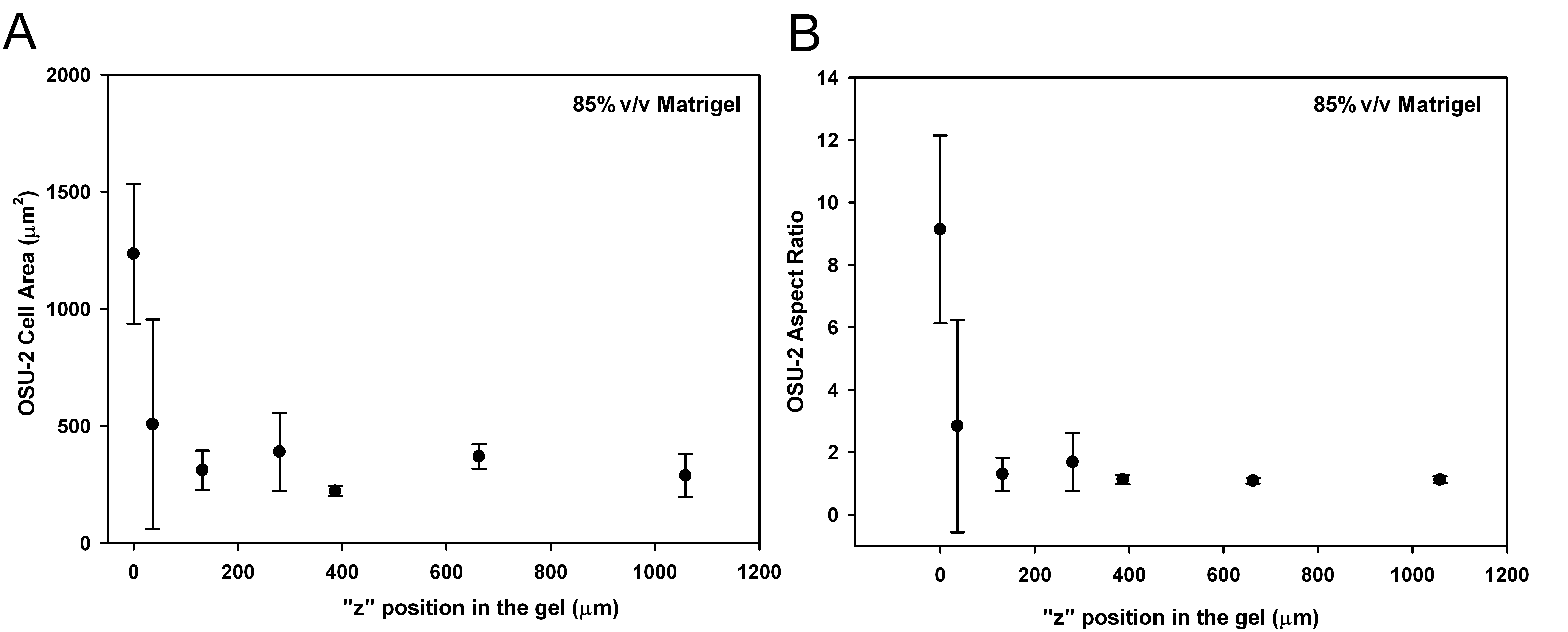

Supplement: Figure S6 — OSU-2 cell morphology quantification. (A) OSU-2 cell area and (B) aspect ratio as a function of observation plane in 85% (v/v) Matrigel. (TIF) [file pone.0035852.s006.tif]

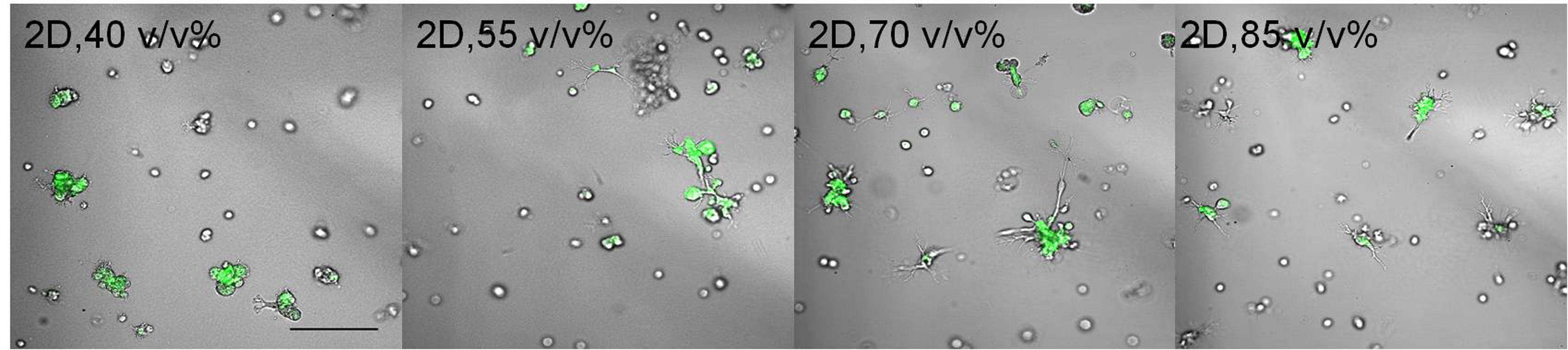

Supplement: Figure S7 — OSU-2 cell morphology in 2D Matrigel for all formulations. Scale bar = 200 µm. (TIF) [file pone.0035852.s007.tif]
